# Prostaglandin E_2_ Inhibits Prostate Cancer Progression by Countervailing Tumor Microenvironment-Induced Impairment of Dendritic Cell Migration through LXR*α*/CCR7 Pathway

**DOI:** 10.1155/2018/5808962

**Published:** 2018-04-04

**Authors:** Kuang Youlin, He Weiyang, Liang Simin, Gou Xin

**Affiliations:** Department of Urology, The First Affiliated Hospital of Chongqing Medical University, Chongqing 400016, China

## Abstract

Migration and homing of dendritic cells (DCs) to lymphoid organs are quite crucial for T cell-induced immune response against tumor. However, tumor microenvironment can make some tumor cells escape immune response by impairing DC migration. Prostaglandin E_2_ (PGE_2_) plays important roles in initiating and terminating inflammatory responses. In this study, we investigated whether PGE_2_ could inhibit murine prostate cancer progression by countervailing tumor microenvironment-induced impairment of dendritic cell migration. We found that murine prostate cancer cell line RM-1-conditioned medium impaired chemotactic movement of marrow-derived DCs and splenic cDCs toward CC chemokine receptor-7 (CCR7) ligand CCL19 in vitro and migration to draining lymph gland in vivo. Meanwhile, it also induced LXR*α* activation and CCR7 inhibition on maturing DCs. However, the treatment of PGE_2_ rescued this impairment of DC migration with upregulation of CCR7 and inhibition of LXR*α*. Further, it was observed that PGE_2_ also increased MMP9 expression and activated Notch1 signaling on DCs. In RM-1-bearing mouse model, PGE_2_ treatment was identified to inhibit tumor growth and induce more tumor-infiltrating T cells and CD11c dendritic cells in tumor sites. Therefore, our findings may demonstrate a new perspective for therapeutic interventions on prostate cancer immunoescape.

## 1. Introduction

Dendritic cells (DCs) are known as the most potent antigen-presenting cells at present and play a central role in tumor-related immune response [1]. Following various antigens such as tumor-associated antigen (TAA) uptake in the peripheral tissue, immature DCs then mature and subsequently move to the secondary lymphoid tissue where they prime T cell response via presenting the antigenic peptides to T-lymphocytes in an MHC-restricted pattern. It was observed that the ability of DCs to initiate an immune response depends on their migration to draining lymph node [2] or immune escape could occur, which frequently is present in growing tumors such as prostate cancer (PCa) [3]. Evidences show that products of lipid and cholesterol metabolism have been demonstrated to cause immunosuppressive effects, including unable to stimulate allogeneic T cells effectively or present TAA as a result of a reduced antigen-processing ability of DCs [4].

Liver X receptors (LXRs) (LXR*α* and LXR*β*), members of the nuclear receptor transcription factor superfamily, are important regulators of cholesterol, fatty acid, and glucose homeostasis that could be activated by oxidized cholesterol (oxysterols) [5]. LXR*β* is expressed ubiquitously, whereas LXR*α* has been detected in the liver, adipose tissue, adrenal glands, the intestine, the lungs, and cells of myelomonocytic lineage [6]. It has been demonstrated that LXRs modulate innate and adaptive immune responses in inflammatory and autoimmune diseases [7]. In addition, studies have reported that LXRs can also promote the elimination of apoptotic cells by DCs and macrophages, thus maintaining immune tolerance [8], and block the proliferation of T and B cells undergoing activation in physiologic conditions [9]. Meanwhile, LXRs can inhibit cancer cell proliferation, which is demonstrated in vitro in plenty of human cancer cells, such as colon and breast cancer cells, T- and B-chronic lymphocytic leukemia (CLL), glioblastoma, and prostate cancer cells [10].

PGE_2_, a metabolite of arachidonic acid, plays important roles in initiating and terminating inflammatory responses [11]. PGE_2_ promotes the ability of DCs to preferentially attract the inhibitory regulatory T cell (Treg) subset of CD4^+^ T cells and to directly improve the development of Tregs [12, 13]. PGE_2_ can also synergize with tumor necrosis factor-*α* (TNF*α*) in the induction of DC maturation and in enhancing CCR7 expression [14]. Based on these observations, PGE_2_ is frequently included in the cytokine cocktails used to produce mature DCs for clinical use as vaccines against cancer [15].

Prostate cancer (PCa) is the most frequently diagnosed cancer in old men and also the second leading cause of male cancer death in the Western countries [16]. Recently, evidence shows that some kinds of cancer, such as melanoma, colon, lung, and kidney tumors inhibit the function of DCs through LXR activation by releasing LXR ligands or oxysterols [17]. Data also indicate that human prostate cancer cells can cause apoptotic death of DCs and markedly inhibit the generation of DCs in cultures [18, 19]. As PGE_2_ plays key roles in inflammation and tumor biology, we aimed to elucidate the role of PGE_2_ on DC migration affected by murine prostate cancer cell line RM-1 and its antitumor effects.

## 2. Materials and Methods

### 2.1. Animals and Cell Lines

Female C57BL/6 (H-2 K^b^) mice, 6–8 weeks old, were obtained from Shanghai SLAC Laboratory Animal Co. Ltd. (Shanghai, China). Animals were maintained at the Central Animal Facility of Chongqing Medical University according to standard guidelines, and experiments were conducted according to the guidelines of the China Council for Animal Care. RM-1, a murine prostate cancer cell line, was obtained from the Chinese Academy of Sciences (Shanghai, China). All cells were cultured in RPMI-1640 medium with 10% FCS, 2 mM L-glutamine, 100 U/ml penicillin, and 100 *μ*g/ml streptomycin at 37°C in a humidified atmosphere containing 5% CO_2_.

### 2.2. DC Generation

Mouse bone marrow-derived DCs were generated from bone marrow suspensions harvested from 6–8-week-old C57BL/6 mice according to the publication [20] with slight modifications. We followed the methods of Youlin et al. [21, 22]. Briefly, bone marrow cells were harvested from femurs and tibias, depleted of red blood cells, and washed twice in PBS. Cells were resuspended in a DC medium consisting of RPMI 1640 supplemented with 10% heat-inactivated fetal calf serum (FCS) (Gibco, America), 10 ng/ml mGM-CSF (R&D Systems, USA), 10 ng/ml mIL-4 (R&D Systems, USA), and 50 mM 2-mercaptoethanol, 100 IU/ml penicillin, and 100 *μ*g/ml streptomycin and cultured (37°C, 5% CO_2_) in 6-well plates at 1 × 10^6^ cells/3 ml/well. On days 3 and 5 of culture, floating cells were gently removed, and fresh mGM-CSF/mIL-4-containing medium was added. On day 6, nonadherent cells and loosely adherent proliferating DC aggregates were collected as immature DCs (iDCs). iDCs were then activated by the inclusion of 10 ng/ml LPS and 1 *μ*g/ml PGE_2_ (Sigma, USA) for another 48 h culture in the presence or absence of conditioned medium from the RM-1.

Spleen DCs were generated as described [23, 24]. Briefly, the spleen tissues were cut into small fragments and digested with collagenase D (Roche, Switzerland). Cells then were centrifuged and resuspended in 5 ml of a 1.077 histopaque (Sigma, USA). An additional 5 ml histopaque was layered below, and the culture medium was layered above the cell suspension, which was then centrifuged. The light density fraction was incubated with the following FITC-conjugated monoclonal antibodies (mAbs) (BD Pharmingen, USA): anti-CD3 (17A2), anti-Thy1.1 (OX-7), anti-B220 (RA3-6B2), anti-Gr-1 (RB68C5), anti-CD49b (DX5), and anti-TER-119 (TER-119). The lineage^−^CD11c^+^ cells were defined as cDCs. The analysis was carried out on a FACS Aria II (Becton Dickinson, San Diego, CA).

### 2.3. siRNA

10 *μ*M LXR-a siRNA (Santa Cruz, California) was added to 300 *μ*l siRNA Transfection Medium (Santa Cruz, California), mixed gently, and incubated at room temperature for 20 min. The scramble siRNA was used as control. The mixture was then added dropwise to the plates with gentle shaking. The transfection media were removed after incubation for 24 h at 37°C, and the cells were transfected again following the same protocol. After another 48 h, the cells were collected for analysis.

### 2.4. Western Blot Analysis

Cell lysates were prepared. Total cellular proteins (50 *μ*g) were subjected to SDS-PAGE and transferred to nitrocellulose membranes (Amersham, USA). Specific polyclonal antibody against LXR*α*, CCR7, ABCG1, ABCA1, MMP9, and Notch1 cleavage (Cell Signaling, Boston, USA) diluted in TBS-T containing 5% nonfat milk was used to detect indicated proteins. The appropriate horseradish peroxidase- (HRP-) conjugated IgG was used as the secondary antibody. An antibody on a membrane was visualized by enhanced chemiluminescence (Pierce, Rockford, IL, USA). Western blot for *β*-actin was used as an internal sample. The quantified densitometry ratio of a target protein to the internal sample was analyzed by Quantity One 4.62 software (Bio-Rad, USA).

### 2.5. Surface Marker Analysis of DCs

For phenotypic analyses by flow cytometry, DCs (5 × 10^5^) were stained for 30 min on ice with FITC- or PE-labeled monoclonal antibodies specific for CD11c, CD80, CD86, and CCR7 (BD Pharmingen). After washing three times in PBS, the cells were analyzed by flow cytometry. Isotype-matched monoclonal antibodies were used as controls.

### 2.6. Transwell Migration Assay

1 × 10^5^ mature DCs were seeded into a transwell (Corning, USA) with a pore size of 5 *μ*m. DCs were allowed to migrate towards chemokine-free RPMI or towards 200 ng/ml CCL19 in RPMI for 3 h at 37°C as described elsewhere [25]. Migrated DCs were harvested from the lower chamber of the transwell and counted by flow cytometry. The percentage of migration was evaluated by the way that the number of migrated DCs was divided by the total number of cells added to the transwell [26].

### 2.7. DC Homing Assay

LPS-activated DCs with conditioned medium from the RM-1 treated or untreated were labeled with 5 *μ*M of carboxyfluorescein diacetate succinimidyl ester (CFSE) (Sigma, USA) in suspension for 10 min at room temperature and then injected (1 × 10^6^) subcutaneously in the hind leg footpad of C57BL/6 mice. 36 h postinjection mice were sacrificed to isolate popliteal lymph nodes and mechanically disaggregated as well as treated with collagenase A (1 mg/ml) and DNase (0.4 mg/ml) mixture in HBSS medium with 20% FBS for 60 min. Single cells were analyzed by flow cytometry as described above.

### 2.8. Tumor Growth in Mouse Xenograft Model

Mice (5 in each group) were shaved on the back and challenged subcutaneously with 2 × 10^5^ RM-1 cells in PBS. PEG_2_ at 100 *μ*l of 0.15 *μ*g *μ*l^−1^ or PBS was then intraperitoneally contralateral every 2 d in total 6 times starting 7d after tumor infusion. Tumor size was evaluated by measuring perpendicular diameters by a caliper. Mice were killed when the tumors displayed severe ulceration or reached a size of 1200 mm^2^.

### 2.9. Immunohistochemistry

Formalin-fixed, paraffin-embedded tissue cancer blocks were cut 4 *μ*m thick sections and mounted on glass slides. After mounting, they were kept in an oven at 70°C for 2 h. Sections were deparaffinized in xylene and rehydrated. Endogenous peroxidase activity was blocked with 3% hydrogen peroxide for 10 min. Antigen retrieval was treated by microwave. Specific polyclonal antibody against CD3 and CD11c diluted in 1% phosphate-buffered saline/bovine serum albumin (1% PBS-BSA) at 1 : 50 was used for incubation overnight. Sections were washed 3 times with PBS and incubated with biotin-labeled IgG for 1 h at room temperature. Then, the sections were stained by a streptavidin-peroxidase detection system (Dako, CA) after 3 washes with PBS. Negative control reactions replaced the primary specific antibody by PBS.

### 2.10. Statistical Analysis

SPSS13.0 was used for data variation analysis. Data are reported as the mean ± SD and were analyzed by the Student *t*-test; *P* values less than 0.05 were considered statistically significant.

## 3. Results

### 3.1. PGE_2_ Downregulates LXR*α* Expression and Activation in Maturing DCs Cultured in RM-1-Conditioned Medium

**Figure 1:**
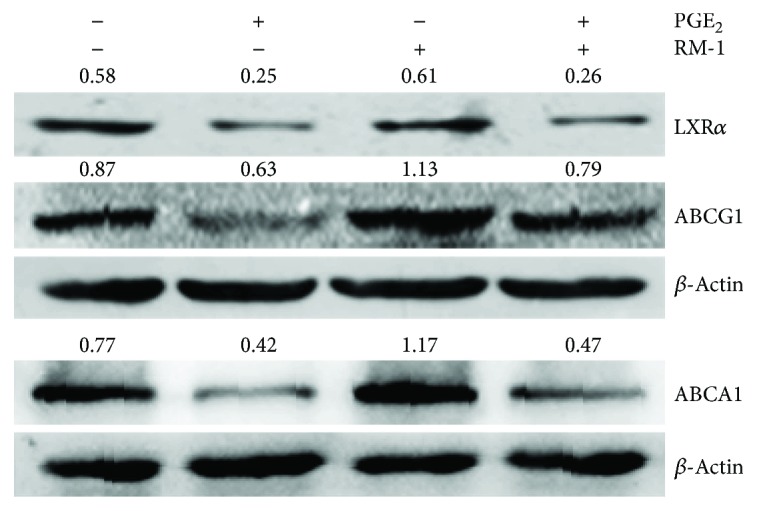
PGE_2_ downregulates LXR*α* expression and activation. The maturing DCs were cultured in the conditioned medium from RM-1. The decrease in ABCG1 and ABCA1 as well as LXR*α* expression by PGE_2_ was detected by Western blot.

Firstly, to determine the effects of prostate cancer cell line RM-1 on LXR*α* expression in maturing DCs, we used the conditioned medium from RM-1 to culture the maturing DCs. The Western blot showed that RM-1-conditioned medium induced LXR*α* activation with increasing the expression of the LXR target gene ABCG1 and ABCA1, while not affecting the expression of LXR*α* in DCs. However, the addition of PGE_2_ reversed this effect by decreasing ABCG1 and ABCA1 expressions and further expression of LXR*α* in DCs (Figure 1).

### 3.2. PGE_2_ Upregulates CCR7 Expression in Maturing DCs Cultured in RM-1-Conditioned Medium via Regulation of LXR*α*

**Figure 2:**
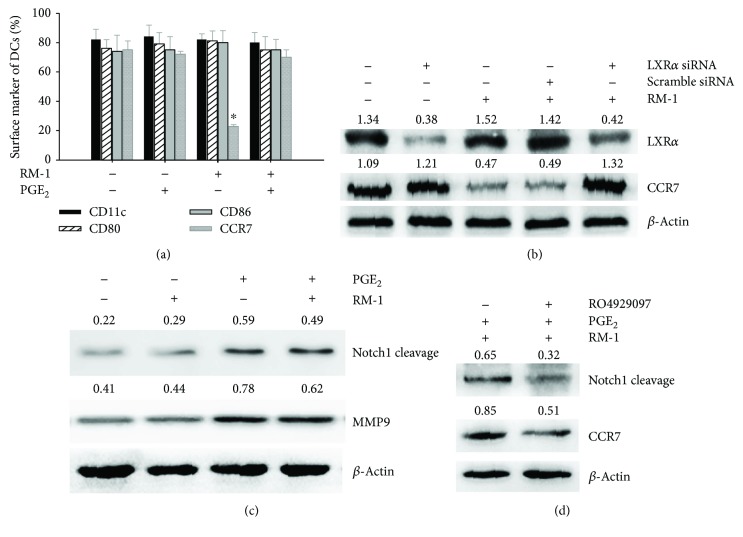
PGE_2_ upregulates CCR7 expression via regulation of LXR*α* and Notch1. (a) Surface markers of CD80, CD86, and CCR7 in maturing DCs cultured in RM-1-conditioned medium were analyzed by flow cytometry. Data were presented as the mean ± SD of 3 independent experiments, each performed in triplicate (^∗^*P* < 0.05). (b) LXR*α* silencing partially reversed the inhibition of CCR7 expression induced by RM-1-conditioned medium in DCs. (c) Increase in expression of MMP9 and Notch1 cleavage by PGE_2_ was analyzed by Western blot. (d) Notch1 cleavage inhibitor RO4929097 partially reversed the inhibition of CCR7 expression induced by RM-1-conditioned medium in DCs using Western blot.

Maturing DCs cultured in RM-1-conditioned medium exhibited lower expression of CCR7, while cytophenotypic markers CD80 and CD86 were upregulated. However, the addition of PGE_2_ reversed this inhibition of CCR7 expression but not that of CD80 and CD86 (Figure 2(a)). To further investigate whether CCR7 could be regulated by LXR*α*, we used LXR*α* siRNA to silence LXR*α* expression. Western blot showed that LXR*α* silencing reversed the inhibition of CCR7 expression induced by RM-1-conditioned medium in DCs (Figure 2(b)). Moreover, we found that PGE_2_ induced increasing expression of MMP9 and Notch1 cleavage (Figure 2(c)), which could be reversed by the addition of *γ*-secretase inhibitor RO4929097 (Figure 2(d)).

### 3.3. PGE_2_ Improves DC Migration In Vitro

**Figure 3:**
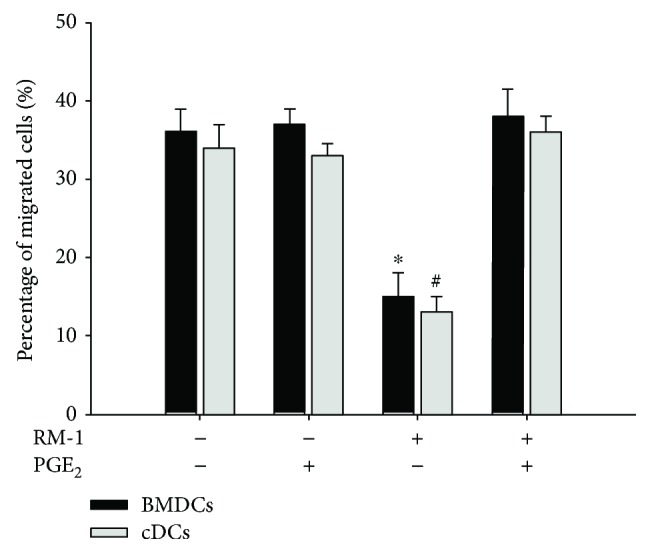
PGE_2_ improves DC migration in vitro. DCs were activated by the inclusion of 10 ng/ml LPS culture in the presence or absence of conditioned medium from the RM-1 as well as PGE_2_. Quantification of the migration of DCs to 200 ng CCL19. Data were presented as the mean ± SD of 3 independent experiments, each performed in triplicate (^∗,#^*P* < 0.05). BMDCs: bone marrow-derived DCs; cDCs: spleen conventional DCs.

Maturing DCs cultured in the presence or absence of conditioned medium from the RM-1 were seeded in the upper reservoirs of transwells, and the number of cells having migrated to the lower reservoir with chemokine CCL19 was assessed by flow cytometry. Data showed that DCs cultured in the RM-1­conditioned medium migrated significantly less efficiently towards the chemokine than that in the absence of RM-1­conditioned medium (Figure 3). However, the addition of PGE_2_ reversed this effect with increasing migrated DCs (Figure 3).

### 3.4. PGE_2_ Promotes DC Homing In Vivo

**Figure 4:**
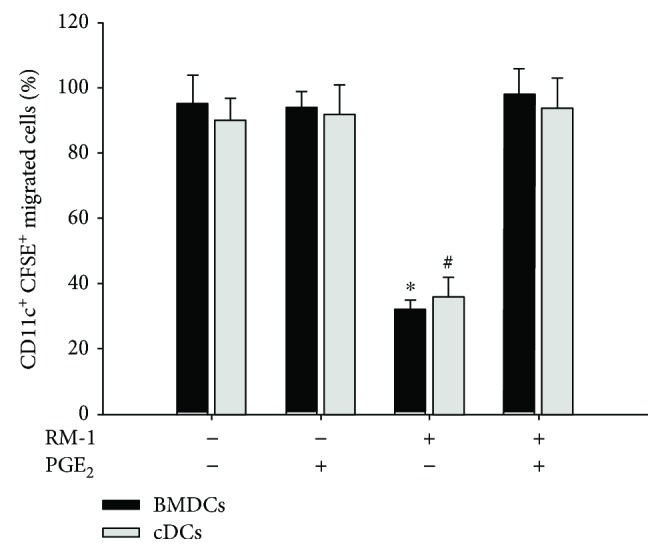
PGE_2_ promotes DCs homing in vivo. DCs were activated by LPS in the presence or absence of conditioned medium from the RM-1 as well as PGE_2_ and stained with CFSE. The percentage of CFSE-positive DCs migrating to the draining inguinal lymph nodes collected from mice injected with DCs was assessed by flow cytometry. Data were presented as the mean ± SD (^∗,#^*P* < 0.05). BMDCs: bone marrow-derived DCs; cDCs: spleen conventional DCs.

To evaluate whether RM-1­conditioned medium impaired DC migration to draining lymph nodes, FACS was used to analyze the draining lymph nodes collected from mice injected with CFSE-stained DCs activated with LPS in the presence or absence of RM-1­conditioned medium. DCs treated with RM-1­conditioned medium migrated poorly to the draining lymph node (Figure 4), but the addition of PGE_2_ reversed this effect with the promotion of DC homing (Figure 4).

### 3.5. PGE_2_ Inhibits RM-1 Cell Growth in Mice with Increased Tumor-Infiltrating T Lymphocytes in Tumor Sites

**Figure 5:**
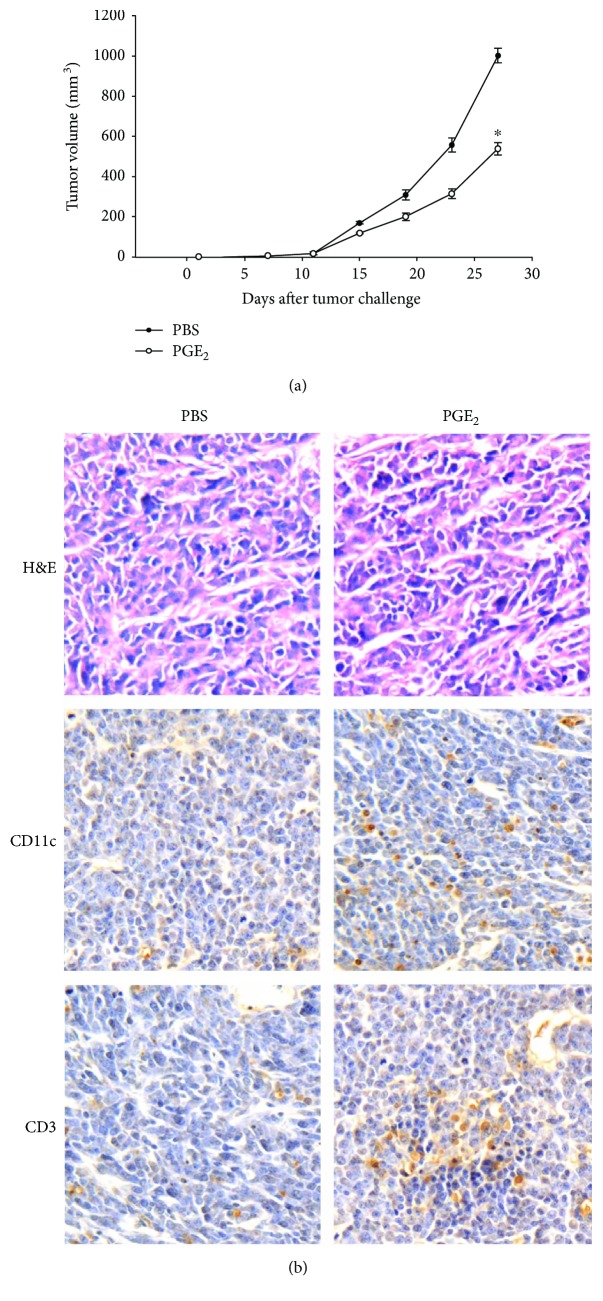
PGE_2_ inhibits RM-1 cell growth in mice with increased tumor-infiltrating T lymphocytes in tumor sites. Mice were challenged subcutaneously with 2 × 10^5^ RM-1 cells. PEG_2_ at 100 *μ*l of 0.15 *μ*g *μ*l^−1^ or PBS was then intraperitoneally contralateral every 2 d in total 6 times starting. (a) The tumor volume was monitored. ^∗^*P* < 0.05. (b) Immunohistochemical analysis of CD3 and CD11c in tumor sections using anti-mouse CD3 and CD11c antibody staining (brown). Original magnification, 400x.

To confirm that the improvement of DC migration by PGE_2_ could further enhance T cell immune response against prostate cancer, RM-1 tumor-bearing mouse models were used for PGE_2_ treating. The result showed that PGE_2_ treatment significantly delayed tumor growth compared to controls (Figure 5(a)). Meanwhile, more tumor-infiltrated CD3^+^ lymphocytes and CD11c^+^ DCs were observed in tumor sites (Figure 5(b)).

## 4. Discussion

DCs are one of the most potent APCs for the induction of antitumor immune responses currently known and due to their strong antitumor effects; DCs emerged as promising candidates for the treatment of PCa patients. Consequently, several clinical trials enrolling PCa patients were conducted, which were based on the administration of DCs pulsed with tumor-associated antigens [27, 28]. However, a number of treated PCa patients were resistant to DC-based immunotherapies, the exact reason of which remains unclear [29]. Here, we found that the conditioned medium from murine prostate cancer cell line RM-1 could inhibit DC migration to draining lymphoid (Figure 4). This impairment of the migratory ability of DCs toward draining lymphoid may result in a reduced antitumor immune response, because DC-induced potent immune response depends on their intact migration from peripheral tissues where they arrest foreign antigens to secondary lymphoid organs where T cell lives [2]. To rescue the migratory ability of DCs, PGE_2_ was added into the RM-1­conditioned medium. We found that PGE_2_ could improve the DC migration, increase tumor-infiltrated T lymphocytes in tumor sites, causing RM-1 tumor-bearing mouse model delayed tumor growth.

PGE_2_ is known to be crucial for immune responses, such as by increasing CCR7-driven DC migration and homing to draining lymph nodes [25, 30], efficient T cell activation [11, 13], keeping the gut mucosal barrier intact against colitis [30], and homeostasis [31]. Here, our study also showed that PGE_2_ reversed the inhibition of CCR7 in DCs from RM-1-conditioned medium (Figure 2(a)) and improved DC migration efficiently to the CCR7 ligand CCL19 (Figure 3). CCR7 is induced together with the maturation of DCs, which was characterized by the upregulated expression of MHC molecules and costimulatory molecules such as CD80, CD83, and CD86, as well as CCR7 [32, 33], and is essential for DC mobilization. The transfer of CCR7-deficient DCs leads to the recovery of less than one-tenth the number of DCs from the lymph node compared with the transfer of CCR7^+^ DCs [34], and DCs differentiated from the bone marrow of CCR7-deficient mice do not move to the draining lymph nodes following their subcutaneous injection or intratracheal instillation [35, 36]. The expression of CCR7 alone, however, is not sufficient for DC migration, sole ligands of which, such as CC-chemokine ligand 19 (CCL19) and CCL21, are essentially involved. Here, we also showed that PGE_2_ upregulated MMP9, known to be important for DC migration [37]. However, the costimulatory molecules such as CD80 and CD86 were not restrained by RM-1-conditioned medium (Figure 2(a)).

Presently, though it is not totally clear how PGE_2_ regulates DC migration on a molecular level, PGE_2_ was shown to improve CCR7 signaling resulting in migration [38]. To further explore how PGE_2_ promotes CCR7 expression, we investigated LXR*α*. We found that RM-1-conditioned medium induced LXR*α* activation with increasing the expression of the LXR target genes ABCG1 and ABCA1. However, PGE_2_ reversed this activation with decreasing ABCG1 and ABCA1 expression and further expression of LXR*α* (Figure 1). Furthermore, after LXR*α* was specifically silenced by LXR*α* siRNA, the inhibition of CCR7 expression was rescued (Figure 2(b)). It demonstrated that PGE_2_ upregulated CCR7 expression in maturing DCs cultured in RM-1-conditioned medium via inhibiting LXR*α*. LXR*α* modulates innate and adaptive immune responses in inflammatory and autoimmune diseases [7]. In addition, data indicate that LXR*α* can also promote the elimination of apoptotic cells by DCs and macrophages, thus maintaining immune tolerance [8], and in vitro differentiation of human DCs in the presence of LXR agonists and LPS has been indicated to influence their T cell stimulatory ability [39]. Moreover, we discovered that the addition of PGE_2_ to RM-1-conditioned medium enhanced the expression of Notch1 cleavage (Figure 2(c)). Additionally, the expression of CCR7 was downregulated when the Notch1 cleavage was inhibited by *γ*-secretase inhibitor RO4929097 (Figure 2(d)). It has been studied that Notch activation upregulates CCR7 expression in leukemic cells [40]. Our data indicated that PGE_2_ preserved the activation of Notch1 signaling in DCs in the presence of RM-1-conditioned medium that were presumably involved in the regulation of CCR7 expression.

## 5. Conclusions

Our findings may demonstrate a possible potential way of prostate cancer immunoescape or immune-tolerance in which prostate cancer impairs DC migration towards draining lymph nodes. At the same way, PGE_2_ may be used as a new perspective for therapeutic interventions on prostate cancer immunoescape.
